# Analysis of Fungal Genomes Reveals Commonalities of Intron Gain or Loss and Functions in Intron-Poor Species

**DOI:** 10.1093/molbev/msab094

**Published:** 2021-03-27

**Authors:** Chun Shen Lim, Brooke N Weinstein, Scott W Roy, Chris M Brown

**Affiliations:** 1 Department of Biochemistry, School of Biomedical Sciences, and Genetics Otago, University of Otago, Dunedin, New Zealand; 2 Quantitative & Systems Biology, School of Natural Sciences, University of California-Merced, Merced, CA, USA; 3 Department of Biology, San Francisco State University, San Francisco, CA, USA

**Keywords:** fungi, intron evolution, intron functions, evolutionary reconstruction, comparative genomics

## Abstract

Previous evolutionary reconstructions have concluded that early eukaryotic ancestors including both the last common ancestor of eukaryotes and of all fungi had intron-rich genomes. By contrast, some extant eukaryotes have few introns, underscoring the complex histories of intron–exon structures, and raising the question as to why these few introns are retained. Here, we have used recently available fungal genomes to address a variety of questions related to intron evolution. Evolutionary reconstruction of intron presence and absence using 263 diverse fungal species supports the idea that massive intron reduction through intron loss has occurred in multiple clades. The intron densities estimated in various fungal ancestors differ from zero to 7.6 introns per 1 kb of protein-coding sequence. Massive intron loss has occurred not only in microsporidian parasites and saccharomycetous yeasts, but also in diverse smuts and allies. To investigate the roles of the remaining introns in highly-reduced species, we have searched for their special characteristics in eight intron-poor fungi. Notably, the introns of ribosome-associated genes *RPL7* and *NOG2* have conserved positions; both intron-containing genes encoding snoRNAs. Furthermore, both the proteins and snoRNAs are involved in ribosome biogenesis, suggesting that the expression of the protein-coding genes and noncoding snoRNAs may be functionally coordinated. Indeed, these introns are also conserved in three-quarters of fungi species. Our study shows that fungal introns have a complex evolutionary history and underappreciated roles in gene expression.

## Introduction

Spliceosomal introns are ubiquitous in eukaryotes. They are removed from all regions of transcripts including the untranslated regions (UTRs) as well as coding sequences (CDS) (De Conti et al. 2013; Shi 2017; Lim et al. 2018). Early studies proposed that introns may be involved in generating multidomain genes by exon shuffling (Logsdon et al. 1995; Patthy 2003; Stoltzfus 2004; Sverdlov et al. 2005), and promoting intragenic recombination for higher fitness (Gilbert 1978; Tonegawa et al. 1978; Comeron and Kreitman 2000; Duret 2001). Notable experimentally supported roles of introns in eukaryotes include: 1) generating protein diversity by alternative splicing (Kempken 2013; Irimia and Roy 2014), 2) harboring noncoding RNA (ncRNA) genes, such as snoRNAs and microRNAs (Chorev and Carmel 2012; Jo and Choi 2015), 3) maintaining genome stability by decreasing the formation of DNA–RNA hybrids called R-loops (Niu 2007; Bonnet et al. 2017), 4) intron-mediated enhancement of gene expression (Niu and Yang 2011; Gallegos and Rose 2015; Laxa 2016; Shaul 2017), 5) harboring binding sites for transcriptional or posttranscriptional regulators of gene expression (Rose 2018), 6) allowing for an additional level of posttranscriptional regulation through regulation of RNA splicing (Witten and Ule 2011), and 7) triggering nonsense-mediated decay in unspliced or partially spliced mRNAs through exon junction complexes (EJCs) (Mekouar et al. 2010; Grützmann et al. 2014; Zhang and Sachs 2015; Hellens et al. 2016). Recently, we have uncovered an unexpected relationship between introns and translation, suggesting a role of 5′-UTR introns in promoting translation of upstream open reading frames (Lim et al. 2018).

The most well-studied introns are those that interrupt the protein-coding regions of genes. Extensive computational studies estimate that the last eukaryotic common ancestor (LECA) had a density of introns of about 4 introns/kb (the number of introns per 1 kb of CDS on average) (Stajich et al. 2007; Csűrös et al. 2011; Koonin et al. 2013; Irimia and Roy 2014). Notably, a study of 99 eukaryotic genomes has revealed a surprising variability of intron densities, ranging from 0.1 introns/kb in the baker’s yeast *Saccharomyces cerevisiae* to 7.8 introns/kb in *Trichoplax adhaerens* (Csűrös et al. 2011). Counterintuitively, *T. adhaerens* is one of the simplest free-living multicellular animals (Srivastava et al. 2008). The large variability of intron densities owes in large part to remarkable differences in rates of intron loss through eukaryotic evolution (Roy and Gilbert 2005; Csűrös et al. 2011) and may, in part, be due to the transposable properties of some spliceosomal introns (Roy 2004; Worden et al. 2009; van der Burgt et al. 2012; Huff et al. 2016; Wu et al. 2017). Several models have also been proposed for the mechanism of intron loss, in particular, through genomic deletion (Loh et al. 2008; Yenerall et al. 2011; Zhu and Niu 2013a) and recombination of cDNA with genomic DNA (Fink 1987; Roy and Gilbert 2005; Zhang et al. 2010; Zhu and Niu 2013b).

As of March 2021, a total of 7,861 fungal genome assemblies were available in NCBI Genome. Fungi and their genomes are of interests for many reasons, notably as food, as plant/animal pathogens/symbionts, and for biotechnology applications (Sapountzis et al. 2015; Wheeler et al. 2017; Chan et al. 2018; Kijpornyongpan et al. 2018; Uhse et al. 2018). Fungi comprise a diverse group of organisms evolving over the past 900 My (Dornburg et al. 2017; Kumar et al. 2017), and this diversity is reflected in diverse histories of exon–intron structures. Some fungal clades have undergone massive loss of introns, in particular, the intracellular parasites microsporidia as well as saccharomycetous yeasts (Byrne and Wolfe 2005; Neuvéglise et al. 2011; Hooks et al. 2014; Corradi 2015; Han and Weiss 2017; Whelan et al. 2019; Priest et al. 2020; Wang et al. 2020). For instance, only 4% of *S. cerevisiae* genes have introns. In contrast, some other fungi, for example, the facultative pathogen *Cryptococcus neoformans*, have a relatively high intron density of 4 introns/kb (Stajich et al. 2007; Csűrös et al. 2011).

Previous results have suggested that frequent intron loss events, relatively few instances of intron gain, and the retention of ancestral introns characterize the evolution of introns throughout most fungal lineages (Csűrös et al. 2007, 2011; Stajich et al. 2007). With thousands of fungal genomes available to date (Priest et al. 2020), it is timely to revisit the ancestral states and scale of intron gain and loss in the fungal kingdom. Our analysis of 644 fungal genomes includes representatives from nearly all phylum-level clades, including the early-diverging Blastocladiomycota, Chytridiomycota, Mucoromycota, Zoopagomycota, Cryptomycota, and Microsporidia phyla. The diversity of exon–intron structures and the wealth of kingdom-wide genomic resources of fungi make them excellent models for studying the intron gain and loss dynamics and the functional roles of introns (Priest et al. 2020). Here, we show how intron gain and loss reshape the exon–intron gene structure and suggest why intron conservation may be important for function.

## Results

### Evolutionary Reconstruction Reveals High Ancestral Intron Densities and a General Bias toward Intron Loss over Intron Gain

**Fig. 1.**
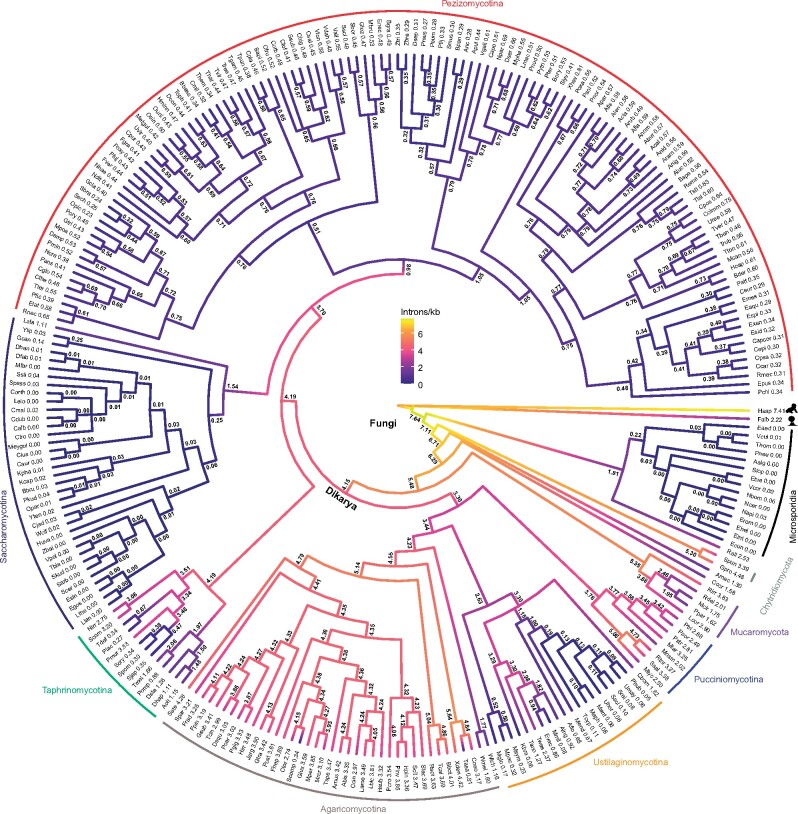
Widespread loss of introns during the evolution of fungi. Ancestral introns were inferred from 1,444 sets of orthologs in 263 fungal species using a Markov model with rates across sites and branch-specific gain and loss rates. Branches are color-coded with intron densities from the median posterior distribution for each node. A list of full names and intron densities are available in supplementary table S2, Supplementary Material online; see also, table 1, figure 2, and supplementary figure S1 and table S1, Supplementary Material online. Green-filled circles denote eight intron-poor species selected for additional analysis. Introns/kb, the number of introns per 1 kb of protein-coding sequence.

**Fig. 2.**
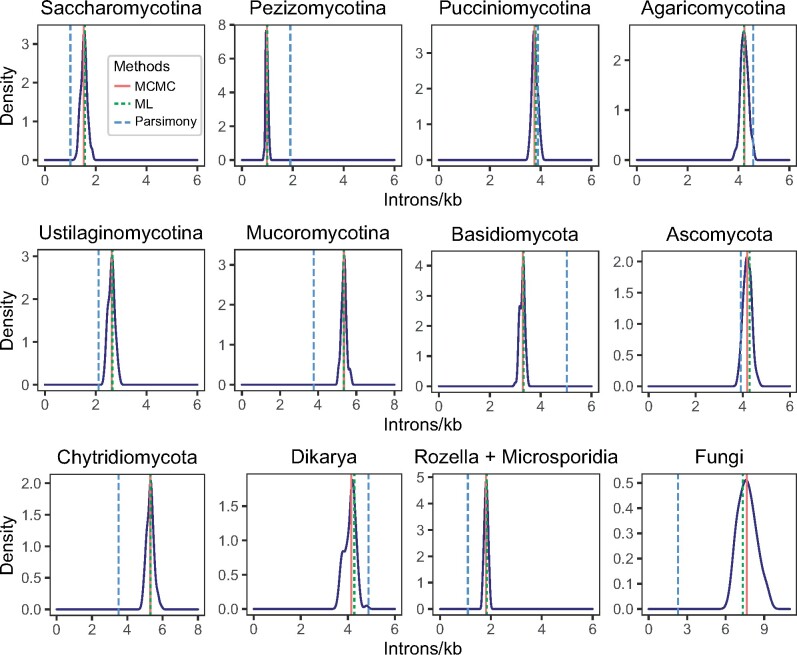
Intron densities of the fungal ancestral states derived from a Monte Carlo approximation of 100 bootstrap distributions. Vertical lines denote the ancestral intron densities inferred from the median values of the 100 MCMC estimates (red), and Dollo parsimony (dotted blue) and maximum likelihood (dotted green) models. The MCMC estimated intron densities of Saccharomycotina, Pezizomycotina, Rozella, and Microsporidia ancestors are lower than 2 introns/kb. In contrast, the MCMC estimated intron densities of Mucoromycotina, Chytridiomycota, and fungal ancestors are higher than 5 introns/kb; see also, figure 1 and table 1. Introns/kb, the number of introns per 1 kb of protein-coding sequence; MCMC, Markov Chain Monte Carlo; ML, maximum likelihood.

We aligned protein sequences and mapped corresponding intron positions for 1,444 sets of orthologous genes from 263 fungal species. We reconstructed the evolutionary history of intron gain and loss among these species. These 263 species represented a wide variety of intron densities, from various intronless Microsporidia to 4.5 introns/kb in the chytrid *Gonapodya prolifera* (fig. 1). This reconstruction revealed a remarkably dynamic and diverse history of intron loss and gain, with many episodes of massive intron loss and/or gain coupled to general stasis within large clades of organisms (e.g., very low intron densities within all Microsporidia and similar intron densities among nearly all Pezizomycotina). Most strikingly, we reconstructed very high ancestral intron densities, with some 7.6 introns/kb reconstructed in the fungal ancestor (fig. 2 and table 1). Although it may be counterintuitive that the ancestral fungus harbored nearly twice as many introns as any modern fungus in this data set, this finding is in keeping with previous results showing a general bias toward intron loss over intron gain in many lineages, and echoes the finding of considerably higher intron densities in alveolate ancestors than in modern alveolates (Csűrös et al. 2011). Although these results are in general agreement with previous studies that inferred intron-rich ancestral fungi (Stajich et al. 2007; Csűrös et al. 2011; Grau-Bové et al. 2017), our inferred densities are considerably higher, likely due to improved model specification made possible by greater species density. Interestingly, our reconstructed value is relatively close to the inferred intron content of the animal ancestor (8.8 introns/kb) in a study using the same reconstruction method on a smaller, eukaryote-wide data set (Csűrös et al. 2011). In contrast to intron-rich ancestral states, almost three-quarters of fungi have evolved to a state of less than 10% of the intron density of the last fungal ancestor (192 of 263 species; fig. 1; see also, supplementary tables S1 and S2 for intron densities at the genomic and orthologous levels, respectively, Supplementary Material online).

**Table 1.** Intron Densities of the Ancestral and Current States of Fungal Clades.

Clade	Number of Species	Ancestral State^a^	Current State^b^	
			Mean	Median
Cryptomycota (*Rozella allomyces*)	1	NA	2.53	2.53
Microsporidia	15	0.22	0.01	0.00
Chytridiomycota	2	5.30	3.93	3.93
Blastocladiomycota (*Allomyces macrogynus*)	1	NA	1.30	1.30
Entomophthoromycotina (*Conidiobolus coronatus*)	1	NA	1.58	1.58
Mucaromycota	5	5.35	2.42	2.01
Pucciniomycotina	8	3.76	2.80	2.74
Ustilaginomycotina	20	2.63	0.51	0.14
Agaricomycotina	39	4.23	3.28	3.41
Taphrinomycotina	13	4.19	1.08	0.88
Saccharomycotina	36	1.53	0.04	0.00
Pezizomycotina	122	0.98	0.47	0.47

Note.—See also figures 1 and 2. Introns/kb, the number of introns per 1 kb of protein-coding sequence; NA, not applicable.

a Obtained from the inference of intron gain and loss.

b Arithmetic mean or median inferred introns/kb of the species within a clade.

These results also illuminate the history of massive intron loss in two lineages. Many studies have found that the obligate intracellular microsporidian parasites have zero or few introns (Keeling et al. 2010; Cuomo et al. 2012; Peyretaillade et al. 2012; Corradi 2015; Desjardins et al. 2015; Han and Weiss 2017; Mikhailov et al. 2017; Ndikumana et al. 2017) and that saccharomycetous yeasts have lost most of their introns (Stajich et al. 2007; Csűrös et al. 2011; Hooks et al. 2014). For both remarkable groups, our analysis includes newly available genomes including relatively intron-rich species at crucial phylogenetic positions (*Rozella allomycis* [2.5 introns/kb], representing a sister lineage to Microsporidia and *Lipomyces starkeyi* [1.1 introns/kb], representing the deepest known branch for Saccharomycotina), allowing for improved resolution of the history of these organisms. In both lineages, our reconstructions reveal a massive intron loss event leading to the ancestor of a large clade of intron-poor organisms. However, whereas in Microsporidia this loss event occurred in the ancestor of the group after divergence from Cryptomycota, for saccharomycetous yeast, this massive loss event occured within the group, after divergence of *L. starkeyi* from the ancestor of all other saccharomycetous yeasts represented in the data set.

### A General Bias toward Intron Loss Punctuated by Several Independent Episodes of Intron Gain

A bias toward intron loss over intron gain is seen across the fungal tree (fig. 1). This is evident not only in Microsporidia and Saccharomycotina but also in groups with more moderate intron densities, including in every species of the filamentous ascomycetes Pezizomycotina and in all smuts/allies within the group Ustilaginomycotina. Indeed, we found a striking bias toward intron loss over gain. Among branches estimated to have undergone at least 5% change in intron density, ten times as many have more loss than gain. Remarkably, a bias is seen even for lineages with very little change (less than 5%), in which intron loss outweighs gain 2-fold (supplementary fig. S1, Supplementary Material online).

Although ongoing intron loss is characteristic of most lineages, our results indicate several substantial episodes of intron gain. Within Basidiomycotina, we estimated a 25% increase in intron density leading to the ancestor of Ustilaginomycetes and a 14% increase in the ancestor of Pucciniomycotina. The most substantial intron gains occurred, unexpectedly, within the famously intron-poor lineages Microsporidia and Saccharomycotina. We inferred substantial, secondary independent intron gain in two extant microsporidians, (*Nosema bombycis* and *Nosema apis*) and four saccharomycetous yeasts (*Scheffersomyces stipitis*, *Candida maltosa*, *Pichia kudriavzevii*, and *Spathaspora passalidarum*). Although preliminary analysis suggests the reality of some of these gains, it is worthy of note that, given the small absolute number of gains involved (leading to <1 intron/kb), further detailed analysis will be necessary to confirm these episodes.

### Intron Density Has a Weak Relationship with Genome Size

Large variations of intron density in fungi raise a question of whether intron density (or number) correlates with genome size. Given that genome size has been argued to relate to the number of introns, organismal complexity, population size, and generation time (Vinogradov 1999; Lynch and Conery 2003; Elliott and Gregory 2015), we examined the relationships between genome size and several features of introns using phylogenetic independent contrasts.

**Fig. 3.**
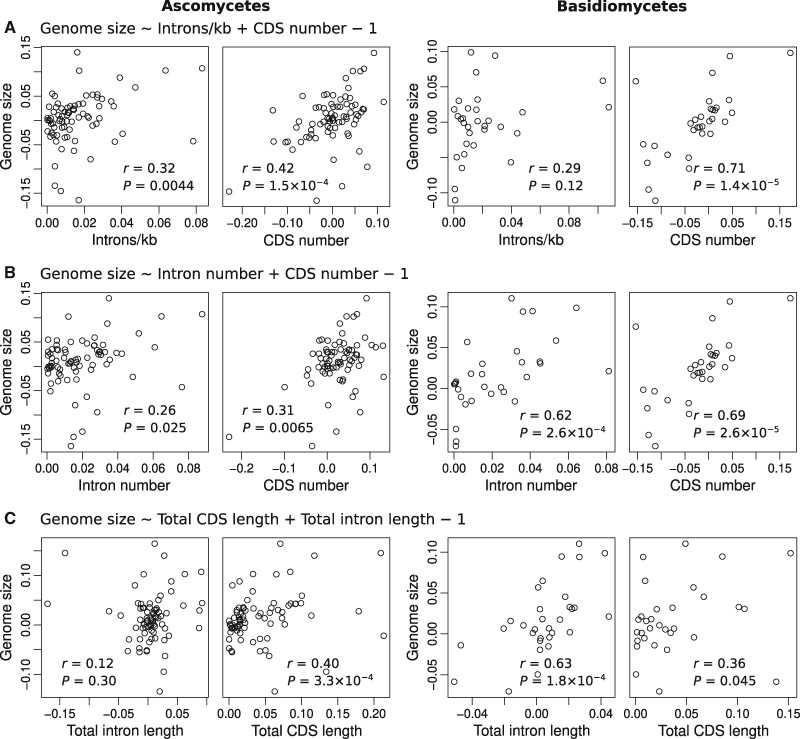
Intron density weakly correlates with genome size in ascomycetes and basidiomycetes. Phylogenetic independent contrasts analysis of (*A*) genome size versus intron density and the number of protein-coding genes, (*B*) genome size versus the numbers of introns and protein-coding genes, and (*C*) genome size versus the total lengths of introns and protein-coding sequences. Data were normalized using Box–Cox transformation prior to this analysis. See also, supplementary figure S2, Supplementary Material online. CDS, coding sequence; *r*, Spearman’s rho.

Interestingly, we found only a weak relationship between genome size and intron density (supplementary fig. S2, Supplementary Material online; Spearman’s rho* *=* *0.26, *P *=* *0.0060). Analyses of ascomycetes and basidiomycetes separately also showed similar results (fig. 3; Spearman’s rho* *=* *0.32 and 0.29, *P *=* *0.0044 and 0.12, respectively), suggesting that this is a common feature of fungi.

In contrast, although the relationship between genome size and intron number is also generally weak across the entire data set (supplementary fig. S2, Supplementary Material online; Spearman’s rho* *=* *0.30, *P *=* *0.0014), this relationship is significantly stronger in basidiomycetes (fig. 3; Spearman’s rho* *=* *0.62, *P *=* *2.6 × 10^−4^). Similarly, total intron length strongly correlates with genome size in basidiomycetes (Spearman’s rho* *=* *0.63, *P *=* *1.8 × 10^−4^). Taken together, these results highlight the distinct characteristics of introns between intron-poor ascomycetes and intron-rich basidiomycetes.

### Intron Gain and Loss Shape the Exon–Intron Gene Structure

**Fig. 4.**
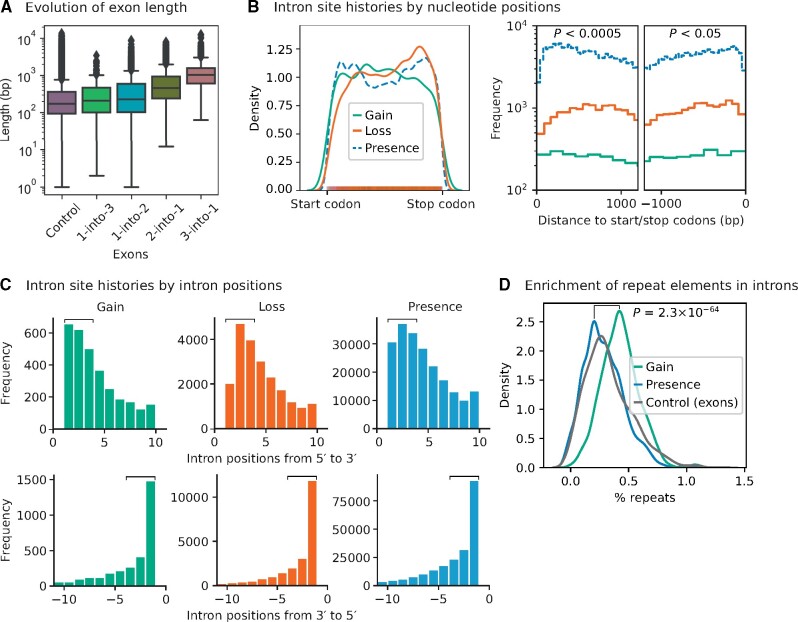
Evolution of the exon–intron gene structure. (*A*) Intron gain and loss (posterior probability ≥0.99) gave rise to significantly larger than average exons. Intron gain resulted in one exon to be split into multiple exons (“1-into-2” or “1-into-3”), whereas intron loss led to a merger of multiple exons (“2-into-1” and “3-into-1”). The median length of “3-into-1” exons is twice larger than “2-into-1,” and six times larger than the extant exons have not been reshaped (“Control”) in 1,444 sets of orthologs (two-sample *t*-tests, *P *<* *10^−170^; see also, supplementary tables S3 and S4, Supplementary Material online). (*B*) Kernel density estimate plot shows the distributions of intron site histories along the gene body (length normalized, left panel). The rug plot superimposes the distributions of intron gain and loss sites. Histograms show the frequencies of intron site histories at the 5′ and 3′ ends of genes (right panel: pairwise Kolmogorov–Smirnov test). (*C*) The frequencies of intron gain, loss, and presence at the first and last three intron positions of genes are significantly heterogeneous (square brackets). Introns are preferentially gained the few intron positions of genes (upper panel: pairwise χ^2^ test for the first three intron positions, *P *<* *10^−8^). In contrast, introns are preferentially lost at the 3′ ends of genes (lower panel: pairwise χ^2^ test for the last three intron positions, *P *=* *5.0 × 10^−44^ for “Loss” vs. “Presence”; *P *=* *4.2 × 10^−6^ for “Gain” vs. “Presence”; *P *=* *0.25 for “Gain” vs. “Loss”). (*D*) Recently gained introns are significantly enriched with repeat elements compared with older introns (Kolmogorov–Smirnov test, *P *=* *2.3 × 10^−64^; see supplementary table S5, Supplementary Material online, for the list of repeat elements detected in recently gained introns). The proportions of repeat elements in introns, exons, and intergenic regions were estimated using bootstrap resampling. The median percent repeats in those introns that are recently gained, presence, and the extant exons have not been reshaped (“Control”) in 1,444 sets of orthologs are 0.42%, 0.28%, and 0.29%, respectively. The median percent repeats in the intergenic regions of 249 or 263 fungi species is 5.4% (supplementary fig. S3, Supplementary Material online).

To explore how gene structures evolve, we computed the lengths of exons that have been reshaped by intron gain or loss events from the analysis of intron/splice site histories (1,444 sets of orthologs). We estimated a false positive rate of 3% for recently gained introns using a posterior probability cutoff of 0.99 (see Materials and Methods, intron site history analysis). We identified four major classes of extant exons that have been reshaped, where one exon has been divided into multiple pieces, that is, 1) “1-into-3” or 2) “1-into-2”, which has arisen from two or one intron gains; and where multiple exons have been fused into one piece, that is, 3) “2-into-1” or 4) “3-into-1”, due to one or two intron losses (fig. 4*A*, see the descriptive statistics in supplementary table S3, Supplementary Material online). Significantly, the median length of “3-into-1” exons is 2-fold larger than “2-into-1” exons, and 6-fold larger than the extant exons that have not been reshaped (two-sample *t*-tests, *P *<* *10^−169^; see also, supplementary tables S3 and S4, Supplementary Material online). This supports previous studies that proposed how intron loss leads to the emergence of extraordinarily large exons (Niu et al. 2005). Curiously, intron gain also produced exons that are significantly larger than older exons (fig. 4*A*, *P *<* *0.05).

We found that intron gain and loss can occur preferentially at different ends of genes (fig. 4*B*). Introns are preferentially gained at the first few intron positions of genes, mainly on genes that previously had a single or two, or no introns (fig. 4*C*, top panel, pairwise χ^2^ tests for the first three intron positions [as indicated by square brackets], *P *<* *10^−8^). In contrast, introns are preferentially lost at the 3′ ends of genes (fig. 4*C*, bottom panel, pairwise χ^2^ test for the last three intron positions [square brackets], *P *=* *5.0 × 10^−44^ for “Loss” vs. “Presence”; *P *=* *4.2 × 10^−6^ for “Gain” vs. “Presence”; *P *=* *0.25 for “Gain” vs. “Loss”), which supports the idea of reverse transcriptase-mediated intron loss (Fink 1987; Roy and Gilbert 2005; Russell et al. 2005; Lee et al. 2010; Zhang et al. 2010; Franzén et al. 2013; Koonin et al. 2013; Zhu and Niu 2013a, 2013b; Irimia and Roy 2014). In addition, we were intrigued by the bimodal position distribution of extant introns (fig. 4*B*), which we have previously observed in some intron-rich metazoa, in particular in *Drosophila melanogaster* (Lim et al. 2018). It would be interesting to study this bimodal position distribution in the future.

Notably, these recently gained introns contain 1.5 times the proportion of repeat elements in older introns (fig. 4*D* and supplementary fig. S3, Supplementary Material online, 0.42% vs. 0.28%; Kolmogorov–Smirnov test, *P *=* *2.3 × 10^−64^). We were able to identify 15 recently gained introns in 12 fungi genomes that harbor repeat elements (supplementary table S5, Supplementary Material online).

These new repeat element-containing introns are likely to be the remnants of transposition episodes that may have occurred millions of years ago. Many transposable elements are no longer active and have degenerated. Nevertheless, we were able to detect many recognizable transposable elements in these recently gained introns, including classes of DNA transposons: 1) Kolobok-H, 2) P Instability Factor (PIF)/Harbinger, and 3) TcMar-Fot1; and retrotransposons: 4) Copia and 5) Gypsy long terminal repeats (LTRs) (supplementary table S5, Supplementary Material online). Notably, we have also found many novel, uncharacterized repeat elements, by predicting repeats in each genome. Further investigation of these novel classes of repeat elements would be useful for understanding the mechanism of intron gain. However, caution is in order here, since it has been found that newly gained introns can secondarily accumulate repetitive sequences (Roy and Penny 2006; Roy 2016).

### Commonalities of Intron-Containing Genes in Intron-Poor Species

Of particular interest are species which retain only a small number of introns, since these introns are likely to be enriched in introns that encode functions. To illuminate the functions of introns in intron-poor species, we chose eight intron-poor species (with intron densities <10% of the fungal ancestor), identified orthologous genes, and analyzed the evolution of intron-containing and intronless genes. These species included *S. cerevisiae* and *Candida dubliniensis* in Saccharomycotina, *Cyphellophora europaea* and *Sporothrix schenckii* in Pezizomycotina, and *Ustilago maydis*, *Pseudozyma hubeiensis*, *Meira miltonrushii*, and *Malassezia sympodialis* in Ustilaginomycotina (fig. 1, green-filled circles), representing six separate massive reductions in intron number.

**Fig. 5.**
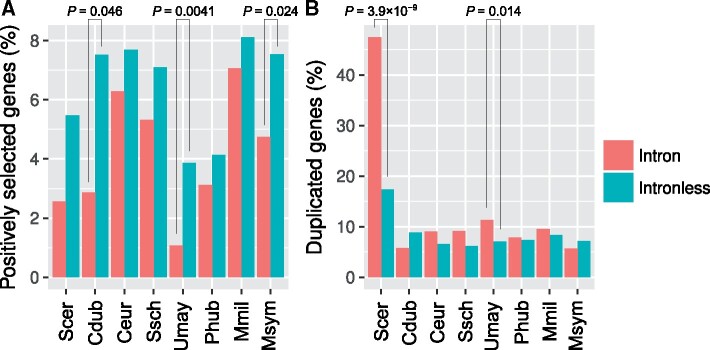
Features of the intron-containing genes in intron-poor fungi. The proportions of intronless and intron-containing genes that have undergone (*A*) positive selection and (*B*) gene duplication, compared using one- and two-sided Fisher’s exact tests, respectively. Cdub*, Candida dubliniensis*; Ceur*, Cyphellophora europaea*; Mmil*, Meira miltonrushii*; Msym*, Malassezia sympodiali*; Phub*, Pseudozyma hubeiensis*; Scer*, Saccharomyces cerevisiae*; Ssch*, Sporothrix schenckii*; Umay*, Ustilago maydis*.

We identified 1,302 complete sets of orthologous genes from these intron-poor species. Comparison of intron-containing genes with intronless genes revealed a number of differences. First, we found that intron-containing genes are less likely to have undergone recent positive selection on their protein-coding sequences than are intronless genes (fig. 5*A*). We propose that this could reflect preferential retention of introns in core genes such as ribosomal protein-coding genes that are less likely to have undergone recent bouts of adaptation.

We also found an association with gene duplication. Significantly higher proportions of the intron-containing genes have duplicate copies in *S. cerevisiae* and *U. maydis*, but not in other species (fig. 5*B*, two-sided Fisher’s exact tests, *P *<* *0.05). In *S. cerevisiae*, this finding could largely be explained by the overrepresentation in highly-expressed ribosomal protein-coding genes. Alternatively, it could largely be explained if intron-mediated cross-regulation among paralogous genes decreases dosage problems associated with gene duplicates (Pleiss et al. 2007; Parenteau et al. 2011; Petibon et al. 2016; Parenteau and Abou Elela 2019). Notably, both of these explanations have key roles for ribosomal protein-coding genes and other genes that date back to the ancestral yeast whole genome duplication; consistent with a crucial role for the genome duplication in establishing this pattern, the association between intron presence and gene duplication is not seen in the related yeast *C. dubliniensis*, which is not descended from the genome duplication. Less is known about *U. maydis*: it would be interesting to see whether the introns of duplicated genes in *U. maydis* are retained through a similar process as that of *S. cerevisiae*.

### Retention of Orthologous Introns in Species with Independent Massive Intron Loss

**Fig. 6.**
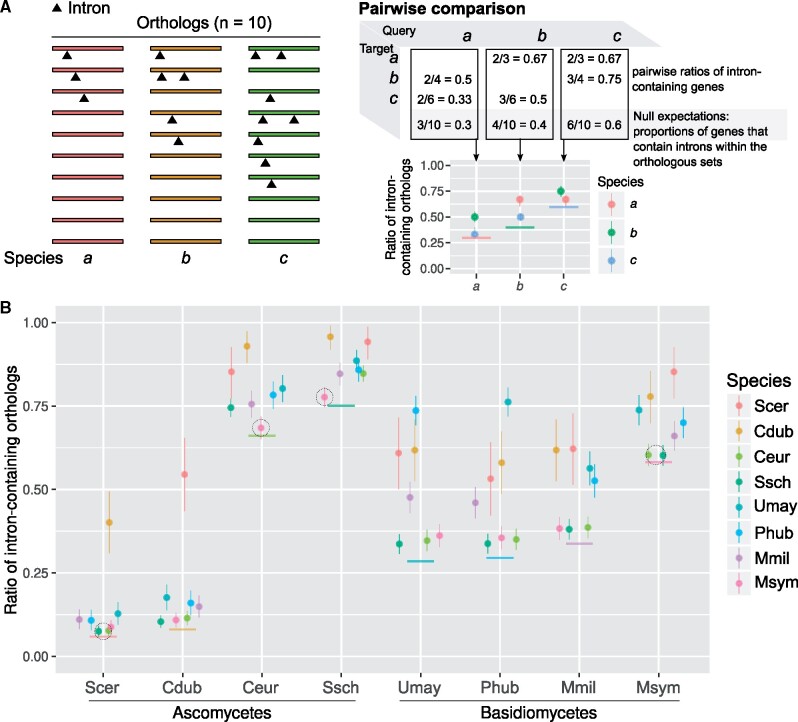
Orthologous genes concordantly harbor introns. (*A*) Schematic example of a pairwise comparison of intron-containing orthologs among three species. (*B*) The ratios of intron-containing orthologs in a pairwise comparison in contrast to null expectations (solid horizontal colored lines). The binomial confidence intervals (95%) were estimated from these ratios using Bayesian inference with 1,000 iterations (vertical colored lines). Dotted circles denote introns may be retained in genes by chance (χ^2^ tests, *P *>* *0.01). Cdub*, Candida dubliniensis*; Ceur*, Cyphellophora europaea*; Mmil*, Meira miltonrushii*; Msym*, Malassezia sympodiali*; Phub*, Pseudozyma hubeiensis*; Scer*, Saccharomyces cerevisiae*; Ssch*, Sporothrix schenckii*; Umay*, Ustilago maydis*.

**Fig. 7.**
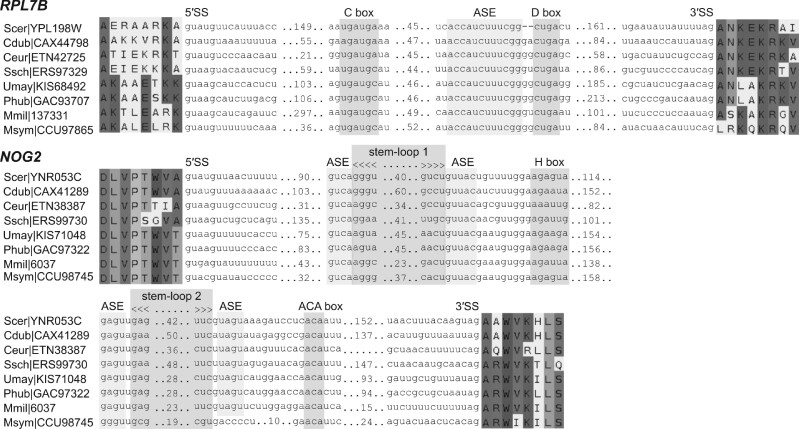
Introns of *RPL7B* and *NOG2* have conserved positions. The introns of *RPL7B* and *NOG2* encode box C/D and box H/ACA snoRNAs (snR59 and snR191 in *S. cerevisiae*, respectively). The predictions of stem-loop 2 and antisense element (ASE) of the *M. miltonrushii* box H/ACA snoRNA are of low confidence. 5′ SS and 3′ SS denote 5′ and 3′ splice-sites, respectively; see also, supplementary figure S4 and table S6, Supplementary Material online. Cdub*, Candida dubliniensis*; Ceur*, Cyphellophora europaea*; Mmil*, Meira miltonrushii*; Msym*, Malassezia sympodiali*; Phub*, Pseudozyma hubeiensis*; Scer*, Saccharomyces cerevisiae*; Ssch*, Sporothrix schenckii*; Umay*, Ustilago maydis*.

If a subset of introns encodes useful functions, we hypothesize that this subset should be preferentially retained in intron-poor species. For each species pair drawn from the eight intron-poor species, we calculated the number of ortholog pairs in which both genes contain introns, and compared this with the null expectation (fig. 6*A*). We found a clear signature toward orthologous genes retaining introns (fig. 6*B*). Strikingly, two orthologous genes—*RPL7B*, which encodes a multifunctional ribosomal protein, and *NOG2*, which encodes a GTPase involved in ribosome biogenesis—have conserved intron positions in all eight intron-poor species (fig. 7). In particular, the *NOG2* intron was previously found to be highly conserved within the family Saccharomycetaceae (Hooks et al. 2014, 2016).

We further explored the conservation of *RPL7B* (or its paralog *RPL7A*) and *NOG2* introns in other fungi species. We found that 82% and 72% of fungi species retain the *RPL7* and *NOG2* intron positions (supplementary table S6 and fig. S4, Supplementary Material online). Interestingly, in *S. cerevisiae*, both *RPL7* and *NOG2* introns contain genes for snoRNAs, specifically a box C/D snR59 (or its paralog snR39) and a box H/ACA snR191, respectively. These noncoding RNAs are involved in rRNA and ribosome biogenesis.

As noted above, none of the fungi species have intron/snoRNA-loss *NOG2* paralogs (supplementary table S6, Supplementary Material online). Only three species have an intron/snoRNA-loss *RPL7* paralog (*Rozella allomycis*, *Trichosporon asahii*, and *Botryobasidium botryosum*). A functional divergence may occasionally occur between the *RPL7* paralogs by allowing intron/snoRNA loss in one paralog.

Our results suggest that these introns with conserved positions have some functions (e.g., as snoRNAs). Interestingly, this conservation may not date to LECA, as snoRNA genes have been shown to move within the genome (Weber 2006; Luo and Li 2007; Schmitz et al. 2008; Hoeppner and Poole 2012). Such dynamics could produce a pattern in which certain introns could be conserved by selection in certain lineages, even large lineages (e.g., all fungi); however, which introns are conserved could be expected to change through evolutionary time, suggesting a pattern of phylogenetic “heterotachy” (Philippe et al. 2005). 

### Roles of Introns in Gene Expression

We observed that introns are closer to initiation codons than the null distributions (supplementary fig. S5, Supplementary Material online), which substantiates the above results (fig. 4) and previous studies (Bon et al. 2003; Mourier and Jeffares 2003; Niu et al. 2005; Russell et al. 2005; Franzén et al. 2013). This observation is consistent irrespective of the roles of intron-containing genes in translation.

**Fig. 8.**
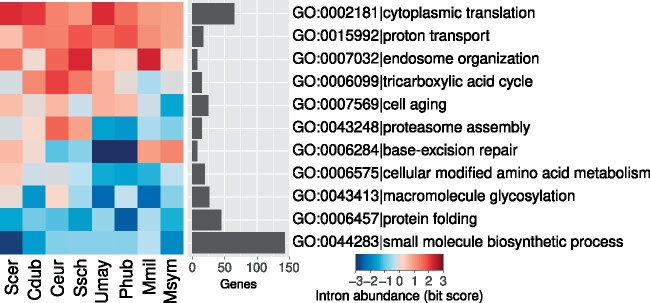
Introns are more abundant in specific classes of genes; see also, supplementary figure S6, Supplementary Material online, for the full results. Cdub*, Candida dubliniensis*; Ceur*, Cyphellophora europaea*; Mmil*, Meira miltonrushii*; Msym*, Malassezia sympodiali*; GO, gene ontology; Phub*, Pseudozyma hubeiensis*; Scer*, Saccharomyces cerevisiae*; Ssch*, Sporothrix schenckii*; Umay*, Ustilago maydis*.

Previous studies have shown that introns are common in the ribosomal protein genes (e.g., *RPL7*) of intron-poor protozoa and saccharomycetous yeasts (Bon et al. 2003; Russell et al. 2005; Franzén et al. 2013). However, the abundance of introns in other classes of genes is less well-known. We examined the gene ontology (GO) terms of the orthologs of the intron-poor species. We found that introns are highly abundant not only in genes involved in cytoplasmic translation (e.g., ribosomal proteins) but also in genes involved in proton transport and endosome organization (fig. 8 and supplementary fig. S6, Supplementary Material online). In contrast, introns are depleted in genes involved in protein folding and small molecule biosynthetic processes. The reasons for these biases are worth further exploration.

These findings prompted us to compare the transcription level and translation efficiency between intron-containing and intronless genes. We analyzed the matched RNA-seq and ribosome profiling data sets for the fungal species that are publicly available—*S. cerevisiae* (Heyer and Moore 2016), *Candida albicans* (Muzzey et al. 2014)*, Schizosaccharomyces pombe* (Subtelny et al. 2014), and *Neurospora crassa* (Yu et al. 2015) (supplementary table S7, Supplementary Material online). We further divided intron-containing and intronless genes by GO terms, that is, with or without the keyword “translation.”

**Fig. 9.**
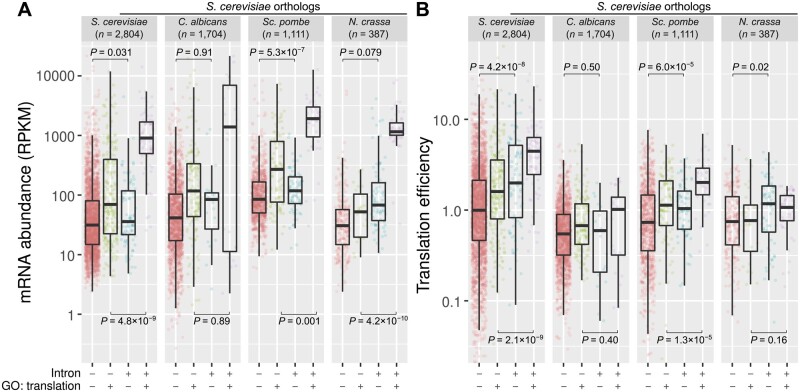
Intron-containing genes have higher levels of mRNA expression and translation efficiency. Matched RNA-seq and ribosome profiling data sets were used in this analysis (supplementary Table S7, Supplementary Material online). (*A*) mRNA abundance was calculated using RPKM. (*B*) Translation efficiency was determined by the ratio of ribosome-protected fragments and RNA-seq read counts that were normalized by the respective library sizes. *Saccharomyces cerevisiae* orthologs were grouped into four classes: (++) intron-containing genes annotated with the GO term “translation,” (+−) intron-containing genes annotated with other GO terms, (−+) intronless genes annotated with the GO term “translation,” and (−−) intronless genes annotated with other GO terms. The levels of mRNA expression and translation efficiency between intron-containing and intronless genes were compared using Welch two-sample *t*-test. *C. albicans*, *Candida albicans*; *N. crassa*, *Neurospora crassa*; GO, gene ontology; RPKM, reads per kilobase per million mapped reads; *S. cerevisiae*, *Saccharomyces cerevisiae*; *Sc. pombe*, *Schizosaccharomyces pombe*.

Notably, intron-containing genes tend to have higher levels of mRNA expression and translation efficiency than intronless genes (fig. 9). This perhaps is not surprising for genes that are associated with translation, in particular for ribosomal protein genes, which are known to be intron-rich and highly expressed (fig. 8). However, we also observed a similar trend for genes that are not associated with translation, suggesting that introns may enhance transcription and translation in both intron-poor and intron-rich fungi. Overall, our results provide independent evidence of diverse roles of fungal introns in transcription and translation.

## Discussion

### A Detailed Portrait of Intron Evolution across a Eukaryotic Kingdom

Eukaryotic species show a huge diversity of exon–intron structures, with massive differences in intron numbers, lengths, and sequences. A large amount of work has probed the origins of these differences. On one hand, some studies have analyzed focused clades of organisms, allowing for detailed insights about the studied clades but raising questions as to the generality of these findings (Roy and Penny 2006; Loh et al. 2008; Sharpton et al. 2008; Zhang et al. 2010; Yenerall et al. 2011; Zhu and Niu 2013b; Hooks et al. 2014; Roy 2016). In addition, such studies may be motivated by preliminary results, raising the possibility that the studied clades are not representative. On the other hand, other studies have compared across widely divergent organisms, attempting to span all of eukaryotic diversity with a relatively small number of deeply-diverged species (Csűrös et al. 2011; Grau-Bové et al. 2017). Such studies allow for the possibility of general conclusions, however the vast evolutionary distances covered raise the spectre of long-branch effects, potentially challenging the statistical conclusions.

In this work, we have leveraged the unprecedented availability of hundreds of genomic sequences spanning a single eukaryotic kingdom. This allows for greater confidence about the generality of our results, given the broad diversity of fungi at various levels from lifestyle, to genome size and complexity to life, and, crucially to intron number and size. This allows us to study many evolutionary branches with minimal change in exon–intron structures, allowing for maximally confident inference, and allows us to compare parallel massive changes in intron number that have occurred within related organisms. These results both provide important confirmations of the generality of trends previously observed and provide several new insights.

### The Diversity of Fungal Exon–Intron Structures

The comparative data set compiled here allows an appreciation of the diversity of fungal exon–intron structures. At one extreme are microsporidian parasites, which have lost all, or nearly all introns. Microsporidian parasites have the smallest eukaryotic genomes and coding capacities known to date (Corradi 2015; Han and Weiss 2017). On the other hand, Chytridiomycota and Mucaromycota, two other early-diverging phyla, are instead characterized by the retention of ancestral introns and thus maintain relatively high intron densities. Indeed, *Gonapodya prolifera*, a chytrid fungus, has the highest intron density of all the fungi in our analysis (4.5 introns/kb), 60% of the intron density of *Homo sapiens*. A great diversity of exon–intron structures is also observed within Dikarya. In general, ascomycetes have roughly half as many introns as do basidiomycetes, however any such generality is belied by substantial diversity within each group. This diversity includes both groups of organisms that are well known to be intron-sparse (Saccharomycotina within ascomycetes, Ustilagomycotina within basidiomycetes) (Byrne and Wolfe 2005; Neuvéglise et al. 2011; Hooks et al. 2014), but also newly-discovered instances of massive intron loss including in the Pezizomycotina fungi *Cyphellophora* and *Sporothrix* sp., conidia producing fungi that have a yeast or yeast-like stage as part of their life cycle (Barros et al. 2011; Feng et al. 2014).

Fungi also show great diversities of intron lengths. At one extreme, the microsporidian parasites that are devoid of introns (e.g., *Anncaliia algerae* and *Nematocida parisii*). At the other, the budding yeast *Candida glabrata* and the smut fungi *Pseudozyma hubeiensis* that harbor long introns (mean* *=* *469 and 426 bp, respectively). Notably, these patterns do not show a simple relationship to intron density. For instance, *S. cerevisiae* has very few introns (*n* = 282), but its introns are relatively long (mean* *=* *397 bp); conversely, the yeast *Cryptococcus neoformans* has many introns (*n* = 34,885) but they are relatively short (mean* *=* *63 bp). Interestingly, though fungi do span most of the known eukaryotic intron density of intron density (see above), they do not reach the extremes of intron length observed elsewhere, for example, mean intron lengths >1 kb in vertebrates.

### A General Trend toward Intron Loss

Previous results have painted a picture in which different lineages experience very different modes of intron evolution, with different lineages experiencing a balance of intron loss over intron gain, or an excess of one over the other (Stajich and Dietrich 2006; Carmel et al. 2007; Worden et al. 2009). Although the evidence collectively suggested that a trend toward intron loss over intron gain was the dominant mode of evolution, limitations existed, including concerns over choice of taxa, and the ability of statistical methods to discriminate intron losses from parallel intron gains. In addition, many studies reconstructed intron loss and gain over very long evolutionary branches, yielding a single ratio of intron losses to gains over potentially very different periods of evolution. The unprecedented species density of our data set allows us to overcome these limitations by reconstruction of intron losses and gains over much shorter timescales, both reducing concerns about statistical errors (since evolutionary reconstruction becomes much more challenging when individual branches represent large amounts of evolutionary change) and allowing for more focused snapshots of the evolutionary process.

We find a remarkable dominance of intron loss over intron gain. Among lineages undergoing at least a 5% change in intron density, 92% have undergone a decrease in intron density. Such a trend may be explained by either selection against introns or mutational bias. That certain other lineages have undergone massive intron gain would seem to weigh against the selective hypothesis. This discrepancy could be explained if such lineages experience altered selective dynamics, for instance, reduced effective population size or reduced selection against nonessential DNA (Lynch 2002; Lynch and Conery 2003). Alternatively, such lineages could experience altered mutational dynamics, fixing intron-creating transposable element insertions (i.e., introner elements) despite their selective costs (Worden et al. 2009; van der Burgt et al. 2012; Huff et al. 2016). Thus the general bias toward intron loss over gain could potentially be explained by general selection against introns.

On the other hand, the bias toward intron loss could largely reflect differences in the rates of mutation. Notably, introners have been found in few eukaryotic lineages, which, together with repeated findings of very infrequent intron gain in many lineages, suggests that only a small fraction of extant eukaryotes harbor active introner elements (a conclusion also supported by an ongoing systematic study by one of us and others). In the absence of introner activity, intron gain appears to occur mostly by highly idiosyncratic events ranging from partial gene duplication to insertion of mitochondrial DNA segments (Li et al. 2009; Curtis and Archibald 2010; Farlow et al. 2011; Hellsten et al. 2011). Perhaps the most productive of these idiosyncratic events is splicing of sequence added by imprecise double-strand break repair (Li et al. 2009; Farlow et al. 2011). However, such events would seem on their face very unlikely to create introns, as they require de novo creation of core and auxiliary splicing signals from a single stochastic event. These considerations suggest that such gains will tend to occur at low rates. Given these considerations, most lineages may experience few intron gains, thus allowing even a very low rate of intron losses to outnumber gains.

### On Methodologies for Reconstructing Intron Loss and Gain

Notably, these results depend on the accuracy of our reconstruction methods. Debate over reconstruction methods for intron loss and gain are nearly as old as the discovery of introns themselves. Concerns have been raised both of overcounting of intron losses and of intron gains (Stajich et al. 2007; Csűrös et al. 2011; Li et al. 2014). Although no methods are perfect, there are two reasons for optimism about the accuracy of the current conclusions. First, limitations of methods generally arise in the context of large amounts of evolutionary change. In particular, when sites for which multiple plausible evolutionary histories exist are common within a data set (for instance, in Rogozin et al.’s classic 8-species data set, an intron position observed in only animals and plants may plausibly be explained by either multiple gains or multiple losses) (Rogozin et al. 2003), choice of reconstruction may depend exquisitely on the evolutionary model (e.g., Buckley 2002; Lartillot et al. 2007). Notably, such effects are particularly pronounced on branches with a high degree of evolutionary change (hence “Long-Branch” effects), and are greatly reduced when the degree of change along individual branches is small. Here, the leveraging of large numbers of relatively closely related species allows us to reconstruct evolution over branches with very small amounts of change (<5% of sites estimated to have undergone changes for >90% of branches in the data set), allowing much more confident reconstruction of specific changes, at least for those branches. Notably, these short branches show a clear trend of an excess of intron losses over intron gains thus affords confidence to the generality of this pattern (fig. 1).

Although the methods used here remain imperfect, previous theoretical and empirical results suggest that the major conclusion, that intron losses tend to outnumber intron gains, is likely to be robust to these concerns. First, previous results have shown that improved taxonomic density tends to increase the estimated ratio of intron losses to intron gains (e.g., compare Csűrös [2005] and Carmel et al. [2007]). Because more speciose data sets contain more information, they are expected to be more accurate, thus the trend toward greater inference of intron loss with improved taxonomic sampling suggests that imperfect data tend to lead to a bias toward intron gains. This is perhaps clearest in the case of reconstruction of the history of vertebrate introns. Initial results suggest massive intron gain in the branch leading from the animal ancestor to modern humans (Csűrös 2005; Nguyen et al. 2005). However, ongoing sampling showed that this inference was incorrect by revealing that the vast majority of human introns are shared with nonbilaterian animals (Sullivan et al. 2006; Srivastava et al. 2008). Accordingly, this result was recovered when data sets were improved to include more slowly-evolving taxa (Grau-Bové et al. 2017). The likely reason for this general bias toward overcounting of parallel intron gains and undercounting of parallel intron losses is described in Stajich et al. (2007). Namely, a failure to account for differences in rates of intron loss between intron sites leads to a failure to reconstruct cases of ancestral introns that have undergone parallel losses from sites experiencing high rates of intron loss. Such failed reconstructions simultaneously undercount ancestral introns and numbers of losses and overcount intron gains, as well as overcounting parallel gains, leading the model to overestimate the incidence of parallel gains, which can have cascading effects on the reconstruction of other sites (Stajich et al. 2007). Thus, we believe that our estimation of intron losses over intron gains as being the major mode of intron evolution is likely to be accurate for the large number of short branches, whereas in the case of long-branch effects, we expect this inference to be robust to methodological concerns.

Also consistent with previous discussions (e.g., Stajich et al. 2007), the least certain parts of the tree are expected to be those that have undergone the most intron gain. Under such circumstances model misspecification will tend to cause multiple intron losses to be incorrectly reconstructed as one or more intron gains. This is of particular importance for the deepest branches within the tree. However, although it may be difficult to determine the precise intron density of some specific ancestors, the considerations directly above suggest that the key biological conclusion, namely that deep fungal ancestors were at least moderately intron-rich, should be robust to these limitations.

Notably, some phylogenetic relationships within fungi remain ambiguous, particularly among early-diverging lineages (James et al. 2020). For our ancestral intron reconstruction, we used a maximum likelihood model (Malin) that requires a fixed species tree (Csűrös 2008). We, therefore, used a preliminary 1,100 taxa version of a concatenated, genome-scale tree (Stajich J, personal communication, December 24, 2018). The final, published version incorporated an additional 500 fungal species (Li et al. 2021) and indeed has some differences in topology between major lineages near the base of the tree. Firstly, the final, more speciose Li et al. (2021) analysis recovered Blastocladiomycota (represented here by *Allomyces macrogynus*) as sister to a clade of Chytridiomycota and fungi. In contrast, we placed Chytridiomycota sister to a clade of Blastocladiomycota and the rest of the fungi. Secondly, the final analysis supported the monophyly of Zoopagomycota (represented here by *Conidiobolus coronatus*) and Mucoromycota. However, the smaller tree we used in our analysis places Zoopagomycota sister to a clade consisting of Mucaromycota and Dikarya. These ancient radiations remain contentious. Both of the alternative groupings used in this analysis were recovered in other studies (e.g., Spatafora et al. 2016; Ahrendt et al. 2018), in addition to receiving intermediate support in the Li et al. (2021) gene tree quartet frequencies. Regardless, our wide taxon sampling within fungi and the relative intron poverty of both *Allomyces macrogynus* and *Conidiobolus coronatus* should limit the impact of potentially misplacing them.

Another ancient relationship within fungi that has been particularly difficult to resolve is that among the three subphyla in Basidiomycota (Prasanna et al. 2020). The concatenated tree used in this study, as well as the larger version in Li et al. (2021), place Pucciniomycotina sister to Ustilaginomycotina and Agaricomycotina/Wallemiomycotina. However, the coalescence analysis in Li et al. (2021) recovered Agaricomycotina as sister to the other two, and gene tree quartet frequencies strongly support each of the three as outgroups (in addition to a hard polytomy). Despite this uncertainty, our overall finding of the maintenance of relatively high ancestral intron densities in Agaricomycotina and Pucciniomycotina versus a substantial loss in Wallemiomycotina and Ustilaginomycotina should be robust to the conflicting evolutionary scenarios.

### Why Do Different Species Have Different Numbers of Introns?

Another major question concerns eukaryotes’ remarkable differences in intron numbers, as reflected in our fungal data set. Previous proposals have emphasized differences in selection on introns in shaping modern intron densities (Lynch 2002; Sun et al. 2015). The notion that mutation rate governs intron density evolution suggests instead that serendipity may play a larger role (Roy and Hartl 2006; Roy and Penny 2007). The amount of intron loss over a given time may largely reflect general evolutionary rates: most lineages will experience intron number reduction, but generally faster evolving lineages will experience greater reduction. Such a pattern is suggested by other studies, in which generally fast-evolving lineages seem to have shed more of their ancestral introns, whereas high intron density in vertebrates seems to almost entirely reflect their atypically slow rate of loss (Denoeud et al. 2010; Venkatesh et al. 2014). Conversely, particularly intron-rich lineages may not be those with atypical selective dynamics, but instead those that happened to have experienced an introner invasion (van der Burgt et al. 2012; Huff et al. 2016). Although differences in selective regime could play a role in which lineages evolve introner elements, it is of note that vertebrates, which have experienced massive invasion of transposable elements, have gained remarkably few introns gains (Venkatesh et al. 2014). Consistent with a lack of a clear role for general selection on genome size or complexity in governing intron loss and gain, we find no strong relationship between intron density and genome size within fungi. Notably, the repeated massive loss of introns in diverse yeasts does indicate a predictability of intron loss. This pattern could be explained by increased selection against introns if unicellular species experience greater selection for rapid replication. However, this pattern could also be explained if the more rapid life cycle of yeasts leads to more cell divisions per unit time and to generally more rapid evolution.

### Why Are There Introns?

What are the functions of introns? To address this question, we chose eight ascomycetes and basidiomycetes with extensive intron loss for in-depth analysis. These intron-poor species all have a yeast or yeast-like stage in their life cycle. Our evolutionary and statistical approaches have shown that remaining introns are unlikely to be conserved by chance (figs. 5–7).

Several studies have shown that the 5′ splice sites of intron-poor species are more conserved than that of intron-rich species (Irimia et al. 2007; Skelly et al. 2009; Neuvéglise et al. 2011). In addition, previous studies have shown that deleting most introns in *S. cerevisiae* does not significantly compromise growth but does compromise starvation resistance (Parenteau et al. 2008, 2011, 2019). These support our idea that only some introns are retained because they encode specific functions.

Our intron conservation analysis prompted us to propose that highly conserved intron positions are indicative of functional importance. For example, the snoRNA genes *snR59* and *snR191* embedded in the introns of *RPL7B* and *NOG2* genes, respectively (fig. 7 and supplementary fig. S4 and table S6, Supplementary Material online). The box C/D snR59 (and its paralog snR39) and the H/ACA snR191 serve as guide RNAs for 2′-O-methylation (A807) and pseudouridylation (Ψ2258 and Ψ2260) of large subunit (LSU) pre-rRNA, respectively (Badis et al. 2003; Piekna-Przybylska et al. 2007). In contrast, RPL7 is required for LSU pre-rRNA processing (27SA_3_ pre-rRNA to 27SB pre-rRNA) (Jakovljevic et al. 2012), whereas NOG2 is involved in cleavaging the C_2_ site of 27SB pre-rRNA, 7S pre-rRNA processing, and the nuclear export of LSU (Saveanu et al. 2001). The conservation of these snoRNA-harboring introns allows the snoRNAs and their host genes to be cotranscribed at high levels and participated in the early and/or middle stage of ribosome biogenesis. In contrast, for snoRNAs that have independent transcription start sites, the expression of several snoRNAs and ribosome protein genes of ascomycetes have been shown to be transcriptionally coregulated (Diao et al. 2014).

Interestingly, a search using a PomBase term “ncRNA in intron” shows four additional protein-coding genes that harbor snoRNAs and two protein-coding genes that harbor ncRNAs in the well-annotated *Sc. pombe* genome (supplementary table S8, Supplementary Material online or https://www.pombase.org/term_genes/PBO:0001137, last accessed November 18, 2020) (Lock et al. 2018). Indeed, some other introns may harbor functional structured RNA elements. For example, the introns of *RPL18A* and *RPS22B* pre-mRNAs that promote RNAse III-mediated degradation, and the *GLC7* intron that modulates gene expression during salt stress (Danin-Kreiselman et al. 2003; Juneau et al. 2006; Parenteau et al. 2008; Hooks et al. 2016).

### Regulatory Roles of Introns in Transcription and Translation

Notably, most of the first introns are located near translation initiation codons (supplementary fig. S5, Supplementary Material online). Indeed, intron loss near the 3′ end of a gene is prevalent in fungi (fig. 4) and some protozoa, probably due to reverse transcriptase-mediated intron loss (Fink 1987; Roy and Gilbert 2005; Russell et al. 2005; Lee et al. 2010; Zhang et al. 2010; Franzén et al. 2013; Koonin et al. 2013; Zhu and Niu 2013a, 2013b; Irimia and Roy 2014).

Introns are also more abundant in ancient genes, in particular, ribosomal protein genes (fig. 8 and supplementary fig. S6, Supplementary Material online). This is in agreement with a previous study on seven saccharomycetous yeasts (Bon et al. 2003). In addition, introns are more abundant in genes that have higher mRNA expression and translation efficiency, irrespective of their cellular functions (fig. 9). This extends previous analyses of global gene expression of *S. cerevisiae* (Juneau et al. 2006). In metazoa and plants, introns may enhance transcription or translation, in part, through EJCs (Wiegand et al. 2003; Diem et al. 2007; Chazal et al. 2013; Le Hir et al. 2016). EJCs deposit at about 20–24 bases upstream of the exon–exon junctions upon splicing, carrying over the “memory” of splicing events to cytoplasmic translation. However, *S. cerevisiae* has no EJCs, unlike complex eukaryotes or even the fission yeast *Sc. pombe*. It remains unclear how intron enhances transcription and translation in *Saccharomycetes* (Moabbi et al. 2012; Hoshida et al. 2017).

### Concluding Remarks

By encompassing an unprecedented number of species and focusing a single group of eukaryotes with a range of very different evolutionary histories, these results allow us to better understand commonalities of intron evolution. We have found a remarkable trend toward intron number reduction across lineages and shown that intron gain and loss produced significantly larger than average exons. We have identified highly predictable patterns of intron retention in intron-poor species at the level of gene function, specific gene, specific intron, and genic position. These characteristics of intron shed light on the potential coordinated functions between genes and introns that warrant further investigation.

## Materials and Methods

### Genome Sequences and Annotations

We retrieved 633 fungal genomes (FASTA and GTF files) from the Ensembl Fungi release 34 (Zerbino et al. 2018). In addition, the *Lipomyces starkeyi* and *Neolecta irregularis* genomes were retrieved from Ensembl Fungi 42 and NCBI Genome, respectively, whereas seven Ustilaginomycotina and two Taphrinomycotina genomes from JGI MycoCosm (Cissé et al. 2013; Grigoriev et al. 2014; Riley et al. 2016; Mondo et al. 2017; Nguyen et al. 2017; Kijpornyongpan et al. 2018). Detailed information can be found in supplementary table S1, Supplementary Material online.

Redundant species were filtered by assembly level (http://ftp://ftp.ncbi.nlm.nih.gov/genomes/ASSEMBLY_REPORTS/assembly_summary_genbank.txt, last accessed December 9, 2016) (Kitts et al. 2016). Complete genomes were retained, otherwise the assemblies at the chromosome, scaffold, or contig levels. For redundant assemblies, only the assemblies with the highest numbers of CDS were retained. A total of 389 genomes passed the quality filters. For outgroups, the genomes of *Homo sapiens* and the cellular slime mold *Fonticula alba* were downloaded from Ensembl 95 and Ensembl Protists 42, respectively.

The annotation of the UTR and UTR introns of *Saccharomyces cerevisiae* was retrieved from YeastMine on April 1, 2017 (Balakrishnan et al. 2012). The GO terms of *S. cerevisiae* were retrieved from the *Saccharomyces* Genome Database on April 27, 2017 (Cherry et al. 2012).

### Taxonomic and Phylogenetic Trees

For the inference of ancestral introns, we manually pruned an 1,100 taxa tree from concatenated analyses (Stajich J, personal communication, December 24, 2018) and retained 263 diverse fungi from 389 quality filtered genomes. *Homo sapiens* and *Fonticula alba* were included as outgroups. For visualization, the tips and nodes were color-coded by inferred intron densities using the R package ggtree v1.16.6 (Yu et al. 2017).

For phylogenetic independent contrasts analysis, we retrieved a timetree from the TimeTree database (http://timetree.org/, last accessed September 19, 2020) using a list of the 263 fungal species and the outgroups (Kumar et al. 2017). This timetree consisted of 113 fungi species for which the evolutionary timescale was available.

### Orthology Analysis

For the inference of ancestral introns, orthologous genes were identified using HMMER v3.1b2 (Johnson et al. 2010). A HMM database was generated concatenating all the precomputed profile hidden markov models (HMMs) from fuNOG (*n* = 19,084 models; eggNOG v4.5) and the 1,000 Fungal Genomes Project (*n* = 434 models; https://github.com/1KFG/Phylogenomics_HMMs/tree/master/HMM/JGI_1086/HMM3, last accessed November 12, 2017) (Huerta-Cepas, Szklarczyk, et al. 2016; Bewick et al. 2019). Homology sequences were detected using hmmsearch. For species that have multiple hits per HMM, only the top hit was retained. To remove false positives, hits with bit scores over 276.48 were retained. This threshold was estimated from the distribution of bit scores (bimodal lognormal) using the R package cutoff v0.1.0 (https://github.com/choisy/cutoff, last accessed July 3, 2019). Specifically, this bimodal distribution was modeled using a finite mixture model whose parameters were estimated by an expectation–maximization algorithm (using the em function with arguments “log-normal,” “normal”), and the threshold was computed by Monte Carlo simulations (using the cutoff function with default settings). Only the orthologs that captured at least 80% (211/265) of the species were used in the subsequent analyses (1,444 sets of orthologs).

Eight intron-poor species were selected for analysis of intron functions, including *S. cerevisiae* and *Candida dubliniensis* in *Saccharomycotina, Cyphellophora europaea* and *Sporothrix schenckii* in Pezizomycotina, and *U. maydis, Pseudozyma hubeiensis, Meira miltonrushii*, and *Malassezia sympodialis* in Ustilaginomycotina. The orthologs of these intron-poor species were identified using proteinortho5 (using parameter -synteny) (Lechner et al. 2011). A total of 1,302 complete sets of orthologs were identified excluding mitochondrial genes. In contrast to the above approach, this approach is less scalable but unrestricted by a predefined set of orthologs (i.e., the HMM database).

Duplicated genes were identified using SkewGD v1 (https://github.com/LongTianPy/SkewGD_v1, last accessed April 24, 2017). This pipeline includes sequence clustering and “age” estimation using *K*_s_ (the number of synonymous substitutions per synonymous site) (Blanc and Wolfe 2004; Vanneste et al. 2013).

### Intron Alignment

For the inference of ancestral introns, protein sequences were aligned using Clustal Omega v1.2.4 (using parameter –hmm-in) (Sievers and Higgins 2018). Annotations of intron positions were extracted from GTF/GFF files using ReSplicer (by calling the splice.extractAnnotations class) (Sêton Bocco and Csűrös 2016). The alignments were realigned using IntronAlignment to improve protein sequence alignments using intron positions while obtaining properly aligned intron sites (Csűrös et al. 2007).

The orthologs of the intron-poor species were aligned using MUSCLE v3.8.31 (Edgar 2004). The protein sequences were realigned using ReSplicer and IntronAlignment as above. To correct mis-annotated intron positions, splice sites were then reannotated using ReSplicer, by calling a series of java classes splice.extractAnnotations, splice.collectStatistics, and splice.checkSites. Realignment was repeated using reannotated intron positions. These intron-aware alignment steps were intended to produce high-quality sequence alignments with properly aligned intron sites.

### Inference of Ancestral Introns

We inferred ancestral introns from 1,444 sets of orthologs of 263 fungal genomes using Malin (Csűrös 2008). Firstly, we generated a table of intron presence or absence in the orthologs using Malin. It included 40,129 intron sites allowing a maximum of 48 ambiguous characters per site. The inference of intron gain and loss rely on unambiguously aligned sequences (Csűrös 2008), meaning that introns around alignment gaps were excluded, at a cutoff of 48 ambiguous characters per intron site. A more robust approach in handling these challenging regions has been proposed (as implemented in ReSplicer), in which the shifts of acceptor- and donor-sites are taken into account in addition to intron gain and loss (Sêton Bocco and Csűrös 2016). However, at the time of writing, this parsimony-based reconstruction approach does not tolerate missing orthologous sequences. We estimated that only 104 of 263 fungi genomes met such criteria, precluding us from using this newer approach.

Failure to account for variation in intron loss rate across sites can lead to an underestimation in intron density of eukaryotic ancestors (Stajich et al. 2007), and previous experiments with rate variation models across sites in Malin showed that model fit was significantly impacted solely by variation in loss rate across intron sites (Csűrös et al. 2011). Here, intron gain and loss rates were optimized in Malin using maximum likelihood with a constant rate and rate-variation model starting from the standard null model and running 1,000 optimization rounds (likelihood convergence threshold* *=* *0.001). For the constant rate model, each intron site has only a branch-specific gain and loss rate. In contrast, for the rate-variation model, intron sites additionally belong to one of two discrete rate loss categories.

Malin calculates gain and loss rates and intron density at the root by numerical optimization of the likelihood. For both the constant rate and rate-variation models, we used 100 bootstrap replicates of the intron table to assess uncertainty about inferred rate parameters and intron site histories for every node. For model comparison, the likelihood-ratio test statistic calculated as:

$$\Delta =-2\times \left(L_{1}-L_{2}\right),$$

where $L_{1}$ is the log-likelihood of the constant rate model ($L_{1}=-354,448$) and $L_{2}$ is the log-likelihood of the rate-variation model ($L_{2}=-349,337$). The likelihood-ratio test statistic is 10,222, which was then compared with a χ^2^ distribution with one degree of freedom. In this comparison, we obtained a *P* value lower than machine precision. Therefore, we rejected the constant rate results and chose the more complex rate-variation model. In addition, we inferred ancestral densities by using Dollo parsimony (Farris 1977).

For all analyses, we scaled the number of inferred introns to intron density by multiplying by 0.30 and dividing by 261, where 0.30 and 261 are intron density and the number of introns in *S. pombe* in the orthologous data set, respectively. *Sc. pombe* was used as a reference because it has a high-quality annotation and over an order of magnitude higher intron density than *S. cerevisiae* (Csűrös et al. 2011; Lock et al. 2018).

### Intron Site History Analysis

Intron site histories were estimated using the rate variation model. Only the intron site histories with a posterior probability ≥0.99 were retained. The candidates of recently gained introns (*n* = 3,328) were filtered by searching against the NCBI Nucleotide database, fungi (taxid: 4751), using BLASTN with default settings (last accessed November 28, 2020). To estimate the false positive rate of recently gained introns, full-length matches to both the query species/genera and the early-branching groups (in the NCBI taxonomy) were considered as false positives and subsequently discarded (*n* = 101).

### De Novo Repeat Family Identification

The repeat families of each fungal genome were identified using Dfam TE Tools v1.2 (Flynn et al. 2020). This de novo repeat family identification pipeline includes RepeatModeler2, RepeatMasker, RepeatScout, coseg, and several other tools (Docker image available at https://github.com/Dfam-consortium/TETools, last accessed September 27, 2020). The repeat families detected were used to build species-specific profile HMM databases using hmmbuild and hmmpress (Johnson et al. 2010) . These databases were used to retrieve the genomic coordinates of repeat elements using dfamscan.pl (bit score threshold* *=* *10) (Hubley et al. 2016).

The overlapping regions between repeat elements and introns, exons, and intergenic regions were obtained using BEDTools v2.27.1 (Quinlan and Hall 2010). The proportions of repeat elements in these genomic features were estimated using bootstrap, that is, resampling 1,000 sets of length matched sequences for 1,000 times. Mitochondrial chromosomes were excluded from this analysis.

### Phylogenetic Independent Contrasts Analysis

The distributions of intron density (introns/kb), genome size, and the number of CDS and introns, and the total lengths of CDS and introns were examined for normality using different transformation functions in the R package bestNormalize v1.6.1. To avoid infinities, a pseudocount of 0.001 was used for intron density, whereas a pseudocount of 1 for intron number and total intron length. Box-cox transformation was chosen and data were transformed using the boxcox function.

Phylogenetic independent contrasts analysis was carried out using the R package caper v1.0.1. Specifically, input variables were mapped to the tips of the timetree phylogeny using the comparative.data function. Independent contrasts (fig. 3) were calculated using the crunch function. Outliers were omitted using the caic.robust function with default settings. A table of contrasts and nodal values were retrieved using the caic.table function.

### Branch-Site Test

The orthologous protein sequences were aligned using PRANK v.150803 (Löytynoja and Goldman 2008; Jeffares et al. 2015). The aligned protein sequences were converted to aligned DNA sequences using PAL2NAL (Suyama et al. 2006). These aligned DNA sequences were used to build phylogenetic trees using RaxML v8.2.9 (using parameters -f a -x 1181 -N 1000 -m GTRGAMMA) (Stamatakis 2014). To identify positively selected genes, branch-site tests were performed using both the aligned DNA sequences and phylogenetic trees using ETE toolkit v3.1.1 (ete-evol, a CodeML wrapper) (Yang 2007; Huerta-Cepas, Serra, et al. 2016). The positive selection (bsA, alternative hypothesis) and relaxation (bsA1, null hypothesis) evolutionary models were fit to the orthologous data set. This involved modeling each branch by recursively marking the remaining branches as the foreground branches, and comparing them using likelihood-ratio tests (using parameters –models M0 bsA bsA1 –leaves –tests bsA, bsA1).

### snoRNA Prediction

The Stockholm alignment files of fungal snoRNA families were downloaded from http://www.bioinf.uni-leipzig.de/publications/supplements.html (last accessed July 5, 2018) (Canzler et al. 2018). These files were used to build HMMs or covariance models using Infernal v1.1.2 (Nawrocki and Eddy 2013). These models were used to detect the snoRNA genes encoded by introns. The functional elements in the snoRNAs were predicted using snoscan v0.2b and the snoGPS web server (Lowe and Eddy 1999; Schattner et al. 2005).

### Gene Ontology Analysis

Functional annotation of *S. cerevisiae* genes was performed using the Bioconductor packages clusterProfiler v3.0.5 and org. Sc.sgd.db v3.4.0 (Yu et al. 2012; Huber et al. 2015). Redundant GO terms were removed using the simplify function of clusterProfiler in conjunction with a semantic similarity cutoff ≤0.5 (Supek et al. 2011). Orthologous genes were grouped by GO terms and the relative intron abundance in a species was calculated as:

$$\mathrm{bitscore}=\log_{2}\left(\frac{x_{i}+1}{s_{x}}\right){-\log}_{2}\left(\frac{c_{i}}{s_{c}}\right),$$

where $x_{i}$ is the number of introns in the genes of species i annotated with the GO term x, $s_{x}$ is the number of genes annotated with the GO term x, $c_{i}$ is the number of introns in the genes of species i within the orthologous sets c, and the number of orthologous sets, $s_{c}=1,030$.

### RNA-Seq and Ribosome Profiling Data Analyses

List of RNA-seq and ribosome profiling data sets used are available in supplementary table S7, Supplementary Material online. The genome and annotation files of *Candida albicans* and *Sc. pombe* were downloaded from the *Candida* Genome Database assembly 22 and PomBase release 30, respectively (Skrzypek et al. 2017; Lock et al. 2018).

Reads were first aligned to ncRNAs using STAR v2.5.2b as previously described (Dobin et al. 2013; Lim et al. 2018). Unmapped reads were then aligned to the genome with transcript annotation. Uniquely mapped reads were counted using featureCounts v1.5.0-p3 (Liao et al. 2014).

The count data of RNA-seq were normalized to reads per kilobase per million (RPKM) mapped reads.

$$\mathrm{RPKM}=\frac{10^{9}\cdot m_{i}}{n\cdot l_{i}},$$

where $m_{i}$ is the number of reads m mapped to gene i, $l_{i}$ is the length l of gene i, and n is the total number of uniquely mapped reads.

For ribosome profiling, translation efficiency (TE) was calculated as:

$$\mathrm{TE}=\frac{p_{i}}{q}\cdot \frac{n}{m_{i}},$$

where $p_{i}$ the number of ribosome footprints p mapped to gene i, q is the total number of uniquely mapped ribosome footprints, $m_{i}$ is the number of RNA-seq reads m mapped to gene i, and n is the total number of uniquely mapped RNA-seq reads.

We detected the *S. cerevisiae* orthologs in other species using proteinortho5 (using parameter -synteny) (Lechner et al. 2011). We found 3,063, 2,506, and 2,541 *S. cerevisiae* orthologs in *C. albicans*, *Sc. pombe*, and *Neurospora crassa*, respectively. The orthologs were grouped by introns presence or absence and GO terms in order to compare their mRNA levels and translation efficiency.

### Statistical Analysis

Statistical analysis and plotting were performed using R v4.0.3 and Python v3.7.7. Fisher’s exact test, χ^2^ test, Welch two-sample *t*-test, Kolmogorov–Smirnov test and Spearman’s rank correlation were calculated using the base R system, SciPy v1.4.1, and Pingouin v0.3.8 (Vallat 2018; Virtanen et al. 2020). Computation of binomial confidence intervals using Bayesian inference was performed using binom v1.1-1. Plots were constructed using ggplot2, Matplotlib v3.1.3, and Seaborn v0.11.0 (Hunter 2007; Wickham 2016), unless otherwise stated. 

## Supplementary Material

Supplementary data are available at *Molecular Biology and Evolution* online.

## Supplementary Material

msab094_Supplementary_Data
