# Supplementary material for: Analysis of Fungal Genomes Reveals Commonalities of Intron Gain or Loss and Functions in Intron-Poor Species
Source: Mol Biol Evol. 2021 Mar 27;38(10):4166–86. doi: 10.1093/molbev/msab094 (PMC8476143; doi:10.1093/molbev/msab094)
Supplement: msab094_Supplementary_Data [file msab094_supplementary_data.zip › Supplementary_Materials_R2.pdf]

Supplementary Materials

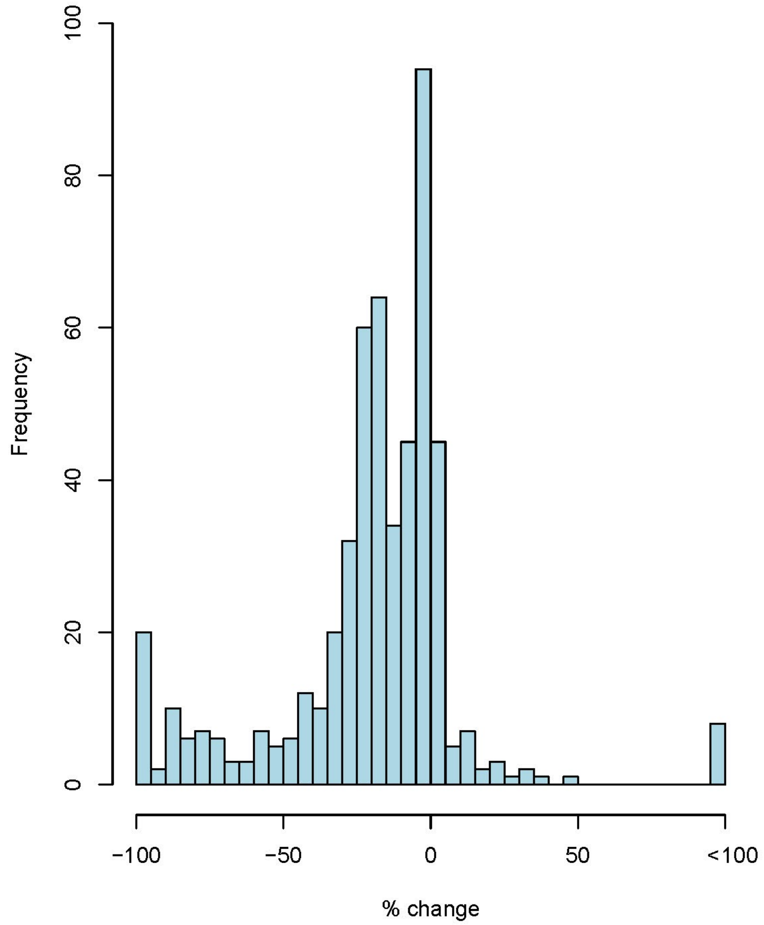

Figure S1. Changes in intron densities along the branches of a fungi taxa tree (Figure 1).

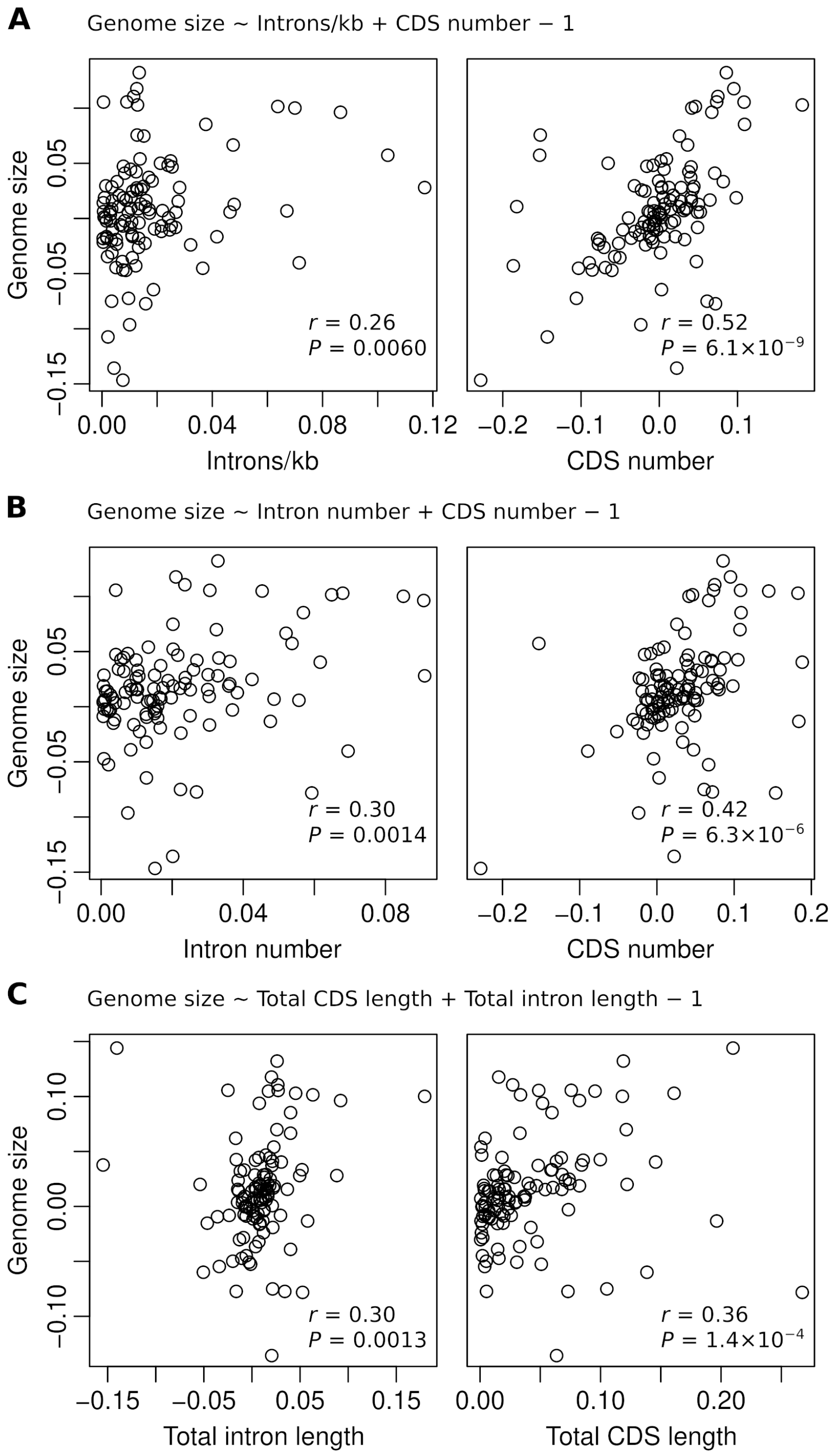

Figure S2. Intron density weakly correlates with genome size in fungi, related to Figure 3. Phylogenetic independent contrasts analysis of **(A)** genome size versus intron density and the number of protein-coding genes, **(B)** genome size versus the numbers of introns and protein-coding genes, and **(C)** genome size versus the total lengths of introns and protein-coding sequences. Data were normalized using Box-Cox transformation prior to this analysis. CDS, coding sequence; *r*, Spearman's rho.

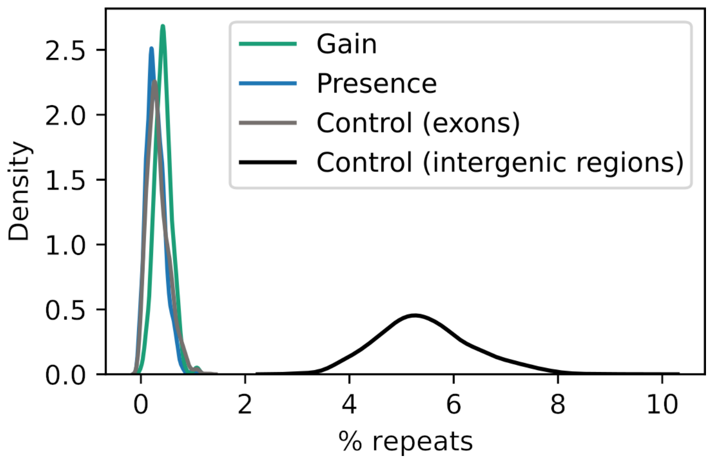

Figure S3. Recently gained introns and intergenic regions are significantly enriched with repeat elements (Kolmogorov-Smirnov tests, $P < 10^{-42}$). Related to Figure 4D and Table S5.

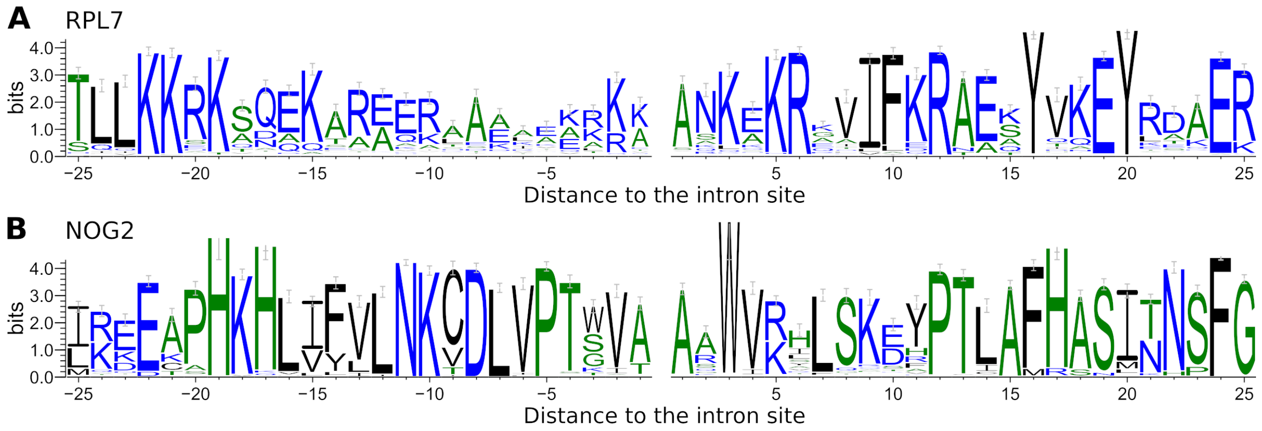

Figure S4. Amino acid sequences flanking the snoRNA harboring introns, related to Figure 7 and Table S6. (A) RPL7 flanking sequences are less conserved than that of **(B)** NOG2.

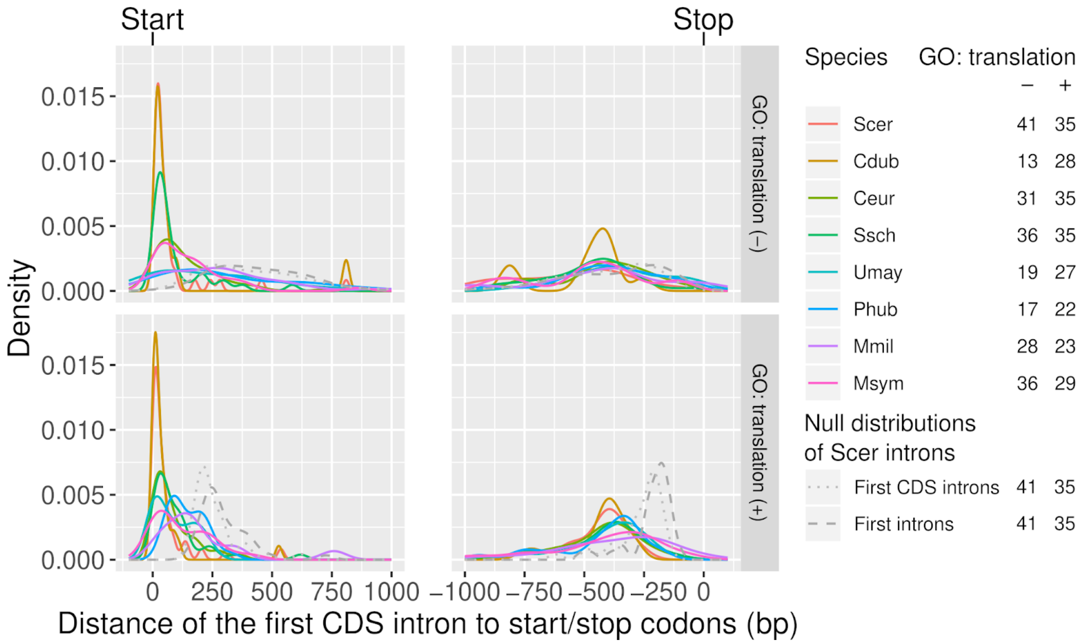

Figure S5. First introns are located near translation initiation codons. Plus and minus signs denote translation-associated genes and other classes of genes, respectively. Gene counts are shown in the right panel. Gray dotted lines denote a null distribution of the first CDS introns of *S. cerevisiae* genes. *S. cerevisiae* is well-annotated and has only one or two introns in its coding genes (GCA_000146045, a total of 282 spliceosomal introns, 273 of 6619 coding genes have introns). Therefore, the expected distances of the first introns from the start codons are either half or one-third of the CDS lengths. Gray dashed lines denote a null distribution of the actual first introns (including UTR introns) of *S. cerevisiae* genes relative to transcription start or termination sites. Genes were split into two groups, i.e. genes related to translation and other classes of genes using gene ontology (GO) terms to account for the distinct intron dynamics in ribosomal protein coding and related genes. CDS, coding sequence; Cdub, *Candida dubliniensis*; Ceur, *Cyphellophora europaea*; GO, gene ontology; Mmil, *Meira miltonrushii*; Msym, *Malassezia sympodiali*; Phub, *Pseudozyma hubeiensis*; Scer, *Saccharomyces cerevisiae*; Ssch, *Sporothrix schenckii*; Umay, *Ustilago maydis*; UTR, untranslated regions.

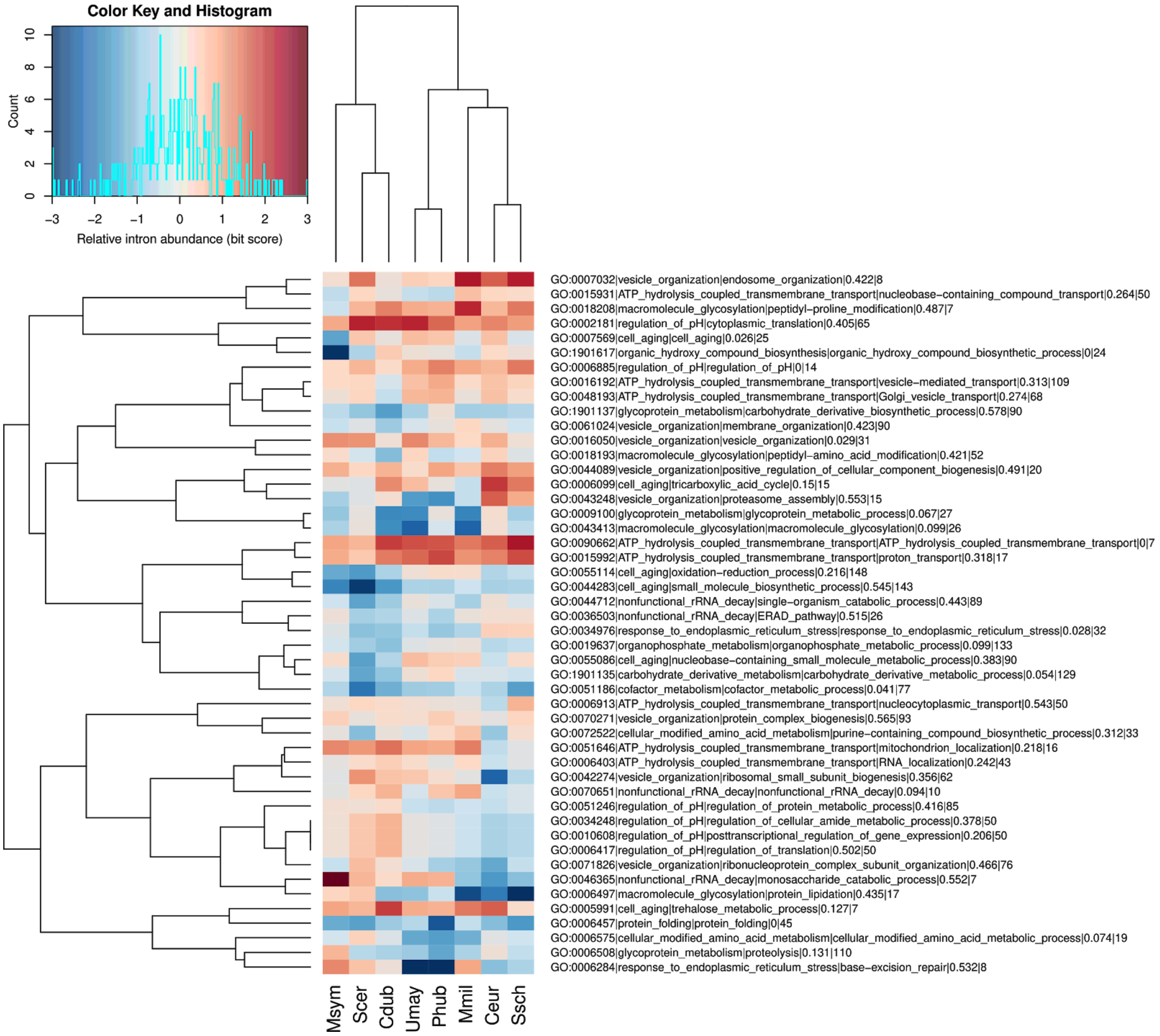

Figure S6. Enrichment of introns in biological processes, related to Figure 8. The y-axis label shows representative GO identifiers, consolidated GO terms, REViGO semantic similarity scores, and the numbers of genes grouped by GO terms. See also Figure 9. Cdub*, Candida dubliniensis*; Ceur*, Cyphellophora europaea*; GO, gene ontology; Mmil*, Meira miltonrushii*; Msym*, Malassezia sympodiali*; Phub*, Pseudozyma hubeiensis*; Scer*, Saccharomyces cerevisiae*; Ssch*, Sporothrix schenckii*; Umay*, Ustilago maydis*.

Table S3. Descriptive statistics of the exons that have undergone intron gain and loss events[a], related to Figure 4A and Table S4.

Exons[b]	Median length (bp)	Counts
1-into-2	229	4092
1-into-3	211	408
2-into-1	462	11864
3-into-1	1045	1254
Control	173	372618

[a] Estimated from 1,444 sets of orthologs using Malin, site histories analysis.
[b] '1-into-3' and '1-into-2' denote the divisions of one exon into three and two pieces, respectively. '2-into-1' and '3-into-1' denote the mergers of two and three exons into one piece, respectively. Most extant exons have not been reshaped ('Control').

Table S4. Pairwise comparison of the lengths of extant exons[a], related to Figure 4A and Table S3.

A[b]	B[b]	T-statistic	Degree of freedom	Unadjusted *P*-value (one-sided)	Bonferroni adjusted *P*-value
2-into-1	3-into-1	-31.247878	1694.000457	6.022856e-170	6.022856e-169
2-into-1	1-into-2	31.361326	5958.437969	2.917357e-200	2.917357e-199
2-into-1	1-into-3	13.925474	426.904630	6.617042e-37	6.617042e-36
2-into-1	Control	102.288538	12777.244768	0.000000e+00	0.000000e+00
3-into-1	1-into-2	48.145953	3295.669566	0.000000e+00	0.000000e+00
3-into-1	1-into-3	25.230827	531.748638	2.993314e-93	2.993314e-92
3-into-1	Control	75.734420	1268.638207	0.000000e+00	0.000000e+00
1-into-2	1-into-3	2.405805	502.299532	8.248630e-03	8.248630e-02
1-into-2	Control	13.846362	4158.325391	5.840735e-43	5.840735e-42
1-into-3	Control	2.107281	407.744897	1.785165e-02	1.785165e-01

[a] Estimated from 1,444 sets of orthologs using Malin, site histories analysis.
[b] '1-into-3' and '1-into-2' denote the divisions of one exon into three and two pieces, respectively. '2-into-1' and '3-into-1' denote the mergers of two and three exons into one piece, respectively. Most extant exons have not been reshaped ('Control').

Table S5. Repeat elements detected in recently gained introns[a], related to Figure 4D and S3.

Species	NCBI taxid	Scaffold/contig	Newly gained intron			Repeat elements
			Start	End	Strand	
Verticillium longisporum	100787	scaffold_1909	357	418	-	DNA/Kolobok-H
	100787	scaffold_361	946	1375	-	DNA/PIF-Harbinger, LTR/Gypsy, Unknown
	100787	scaffold_5	1003	5976	-	Unknown
Fistulina hepatica	1128425	FISHEscaffold_96	5251	5308	+	LTR/Gypsy
Termitomyces sp.	1306850	scaffold257	5162	5213	+	LTR/Gypsy
Rasamsonia emersonii	1408163	ctg1118621762105	243	361	-	DNA/TcMar-Fot1, Unknown
Verruconis gallopava	253628	supercont1.23	5661	5718	-	LTR/Gypsy, Unknown
Puccinia sorghi	27349	Scaffold_1612	834	893	+	LTR/Gypsy, Unknown
Coccidioides immitis	454286	DS268185	81	144	+	Unknown
Fusarium fujikuroi	5127	seq_23	50	99	-	Unknown
Schizophyllum commune	578458	SCHCOscaffold_10	9955	10010	-	Unknown
Nosema bombycis	578461	NBO_591	152	229	-	Unknown
Dothistroma septosporum	675120	DOTSEscaffold_6	9879	9950	+	Unknown
Ophiocordyceps sinensis	911162	OCS_Scf_124	471	527	+	LTR/Copia, Unknown
	911162	OCS_Scf_124	685	753	+	LTR/Copia, Unknown

[a] Estimated from 1,444 sets of orthologs using Malin, site histories analysis.

Table S6. Conservation of snR59/snR39 (box C/D) and snR191 (box H/ACA) locations in *RPL7* and *NOG2* introns in 263 diverse fungi, respectively. Related to Figure 7 and S4.

	Percent species (fraction)	
	snR59/snR39	snR191
Presence	94% (246/263)	95% (249/263)
Conserved intron position	82% (201/246)	72% (170/249)
Two paralogs (snoRNA and host genes)	0.060% (12/201)	0.018% (3/170)
Three paralogs (snoRNA and host genes)	0.010% (2/201)	0% (0/170)
Intron/snoRNA-loss paralogs	0.015% (3/201)[a]	0% (0/170)

[a]Losses occurred in *Rozella allomycis* (taxid: 988480), *Trichosporon asahii* (taxid: 1220162), and *Botryobasidium botryosum* (taxid: 930990).

Table S7. Datasets used for RNA-seq and ribosome profiling analysis, related to Figure 9.

Species	RNA-seq	Ribosome profiling	Adapter sequence (5′-3′)	PMID
S. cerevisiae	SRR3029404, SRR3029405	SRR3029398, SRR3029399, SRR3029400, SRR3029401	TGGAATTCTCGGGTGCCAAGG	26871635
C. albicans	SRR1027797, SRR1027798	SRR1027794, SRR1027795, SRR1027796	CTGTAGGCACCATCAAT	24732588
Sc. pombe	SRR1039856	SRR1039857	-	24476825
N. crassa	SRR2105978	SRR2105977	AGATCGGAAGAGCACACGTCT	26321254
